# Supplementary material for: Graph Theory-Based Electroencephalographic Connectivity via Phase-Locking Value and Its Association with Ketogenic Diet Responsiveness in Patients with Focal Onset Seizures
Source: Nutrients. 2022 Oct 23;14(21):4457. doi: 10.3390/nu14214457 (PMC9659238; doi:10.3390/nu14214457)
Supplement: Supplementary file 1 [file nutrients-14-04457-s001.zip › Table S1.pdf]

Table S1. CBCL scores at initiation and at 12 months after the initiation of ketogenic diet therapy.

	Father report (n=10)					Mother report (n=13)						
	Initiation Mean ± STD	12 months Mean ± STD	Diff	Ratio (%)	*p*¶	Initiation Mean ± STD	12 months Mean ± STD	Diff	Ratio (%)	*p*£	*p*¢	*p*¥
Syndrome Scale Scores												
Anxious / Depressed	52.10 ± 3.51	52.70 ± 3.47	0.60 ± 3.63	1.36 ± 6.79	0.61	55.38 ± 7.30	53.77 ± 5.92	-1.62 ± 6.98	-2.07 ± 10.85	0.40	0.56	0.50
Withdrawn / Depressed	58.70 ± 8.03	56.50 ± 5.58	-2.20 ± 6.29	-2.86 ± 10.09	0.37	59.31 ± 7.86	57.62 ± 8.40	-1.69 ± 5.28	-2.60 ± 8.84	0.36	0.95	0.98
Somatic Complaints	54.80 ± 6.36	55.30 ± 6.62	0.50 ± 6.57	1.48 ± 12.69	0.46	60.31 ± 9.76	54.23 ± 5.25	-6.08 ± 7.34	-8.84 ± 10.49	**0.02***	0.10	0.10
Social Problems	59.20 ± 7.80	57.30 ± 5.70	-1.90 ± 6.62	-2.32 ± 10.96	0.36	61.23 ± 7.73	59.62 ± 6.92	-1.62 ± 7.49	-1.82 ± 12.32	0.35	0.64	0.62
Thought Problems	56.70 ± 7.39	55.30 ± 6.17	-1.40 ± 8.03	-1.51 ± 12.86	0.60	59.38 ± 8.58	58.31 ± 8.97	-1.08 ± 5.38	-1.55 ± 9.10	0.37	0.90	0.92
Attention Problems	61.70 ± 13.94	56.10 ± 6.64	-5.60 ± 12.78	-6.46 ± 16.17	0.34	62.92 ± 8.23	61.08 ± 9.33	-1.85 ± 6.05	-2.78 ± 9.63	0.26	0.98	0.90
Rule-Breaking Behavior	52.40 ± 3.31	53.10 ± 3.45	0.70 ± 2.87	1.46 ± 5.57	0.75	52.31 ± 2.78	53.15 ± 4.18	0.85 ± 4.28	1.75 ± 8.02	0.68	0.68	0.66
Aggressive Behavior	53.60 ± 5.42	54.20 ± 6.68	0.60 ± 6.17	1.42 ± 10.59	0.75	56.31 ± 7.22	53.23 ± 6.33	-3.08 ± 5.87	-4.93 ± 8.67	0.05	0.11	0.11
DSM-Oriented Scales												
Depressive Problems	61.10 ± 8.01	58.90 ± 7.26	-2.20 ± 7.60	-2.86 ± 11.41	0.67	65.62 ± 7.83	60.77 ± 9.95	-4.85 ± 5.98	-7.45 ± 9.08	**0.02***	0.25	0.23
Anxiety Problems	55.00 ± 6.31	53.10 ± 4.43	-1.90 ± 7.48	-2.49 ± 12.29	0.62	57.31 ± 9.20	55.00 ± 6.51	-2.31 ± 7.36	-2.81 ± 11.99	0.44	1.00	1.00
Somatic Problems	53.70 ± 5.70	54.40 ± 6.08	0.70 ± 7.56	2.03 ± 13.91	0.92	56.15 ± 10.14	52.46 ± 4.03	-3.69 ± 9.02	-4.70 ± 12.76	0.23	0.36	0.36
Attention Deficit/hyperactivity disorder	58.70 ± 9.80	55.10 ± 5.67	-3.60 ± 10.49	-4.18 ± 15.59	0.40	58.85 ± 7.19	55.85 ± 5.61	-3.00 ± 4.71	-4.63 ± 7.38	0.05	0.58	0.58
Oppositional Defiant Problems	53.90 ± 6.71	53.90 ± 7.05	0.00 ± 6.34	0.40 ± 9.61	1.00	56.69 ± 7.92	53.77 ± 6.10	-2.92 ± 6.97	-4.30 ± 10.02	0.23	0.51	0.51
Conduct Problems	52.80 ± 4.87	52.10 ± 3.51	-0.70 ± 6.02	-0.68 ± 10.18	0.89	54.08 ± 5.63	53.31 ± 5.34	-0.77 ± 7.81	-0.54 ± 13.46	0.67	0.90	0.90

$^{*}p < 0.05$; STD: Standard deviation; Diff: 12 months - initiation; Ratio: (12 months - initiation)/ initiation; *p*¶: Father (12 months vs. initiation), Wilcoxon Signed Ranks Test; *p*£: Mother (12 months vs. initiation), Wilcoxon Signed Ranks Test; *p*¢: 12 months – initiation, (Father vs. Mother), Mann-Whitney U; *p*¥: (12 months - initiation)/initiation, (Father vs. Mother), Mann-Whitney U.
