# Supplementary figures and images for: Graph Theory-Based Electroencephalographic Connectivity via Phase-Locking Value and Its Association with Ketogenic Diet Responsiveness in Patients with Focal Onset Seizures
Source: Nutrients. 2022 Oct 23;14(21):4457. doi: 10.3390/nu14214457 (PMC9659238; doi:10.3390/nu14214457)

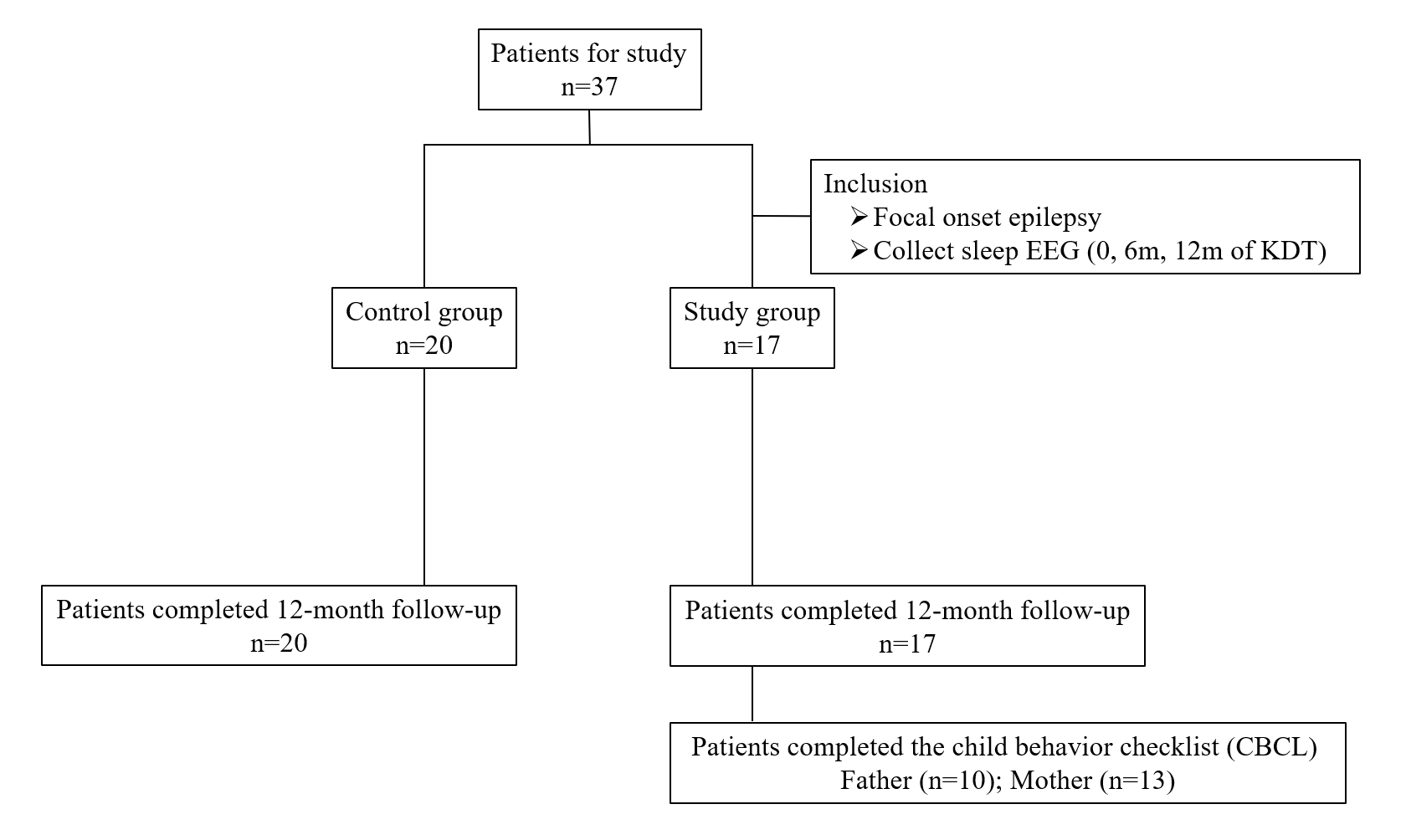

Supplement: Supplementary file 1 [file nutrients-14-04457-s001.zip › Figure S1.tif]

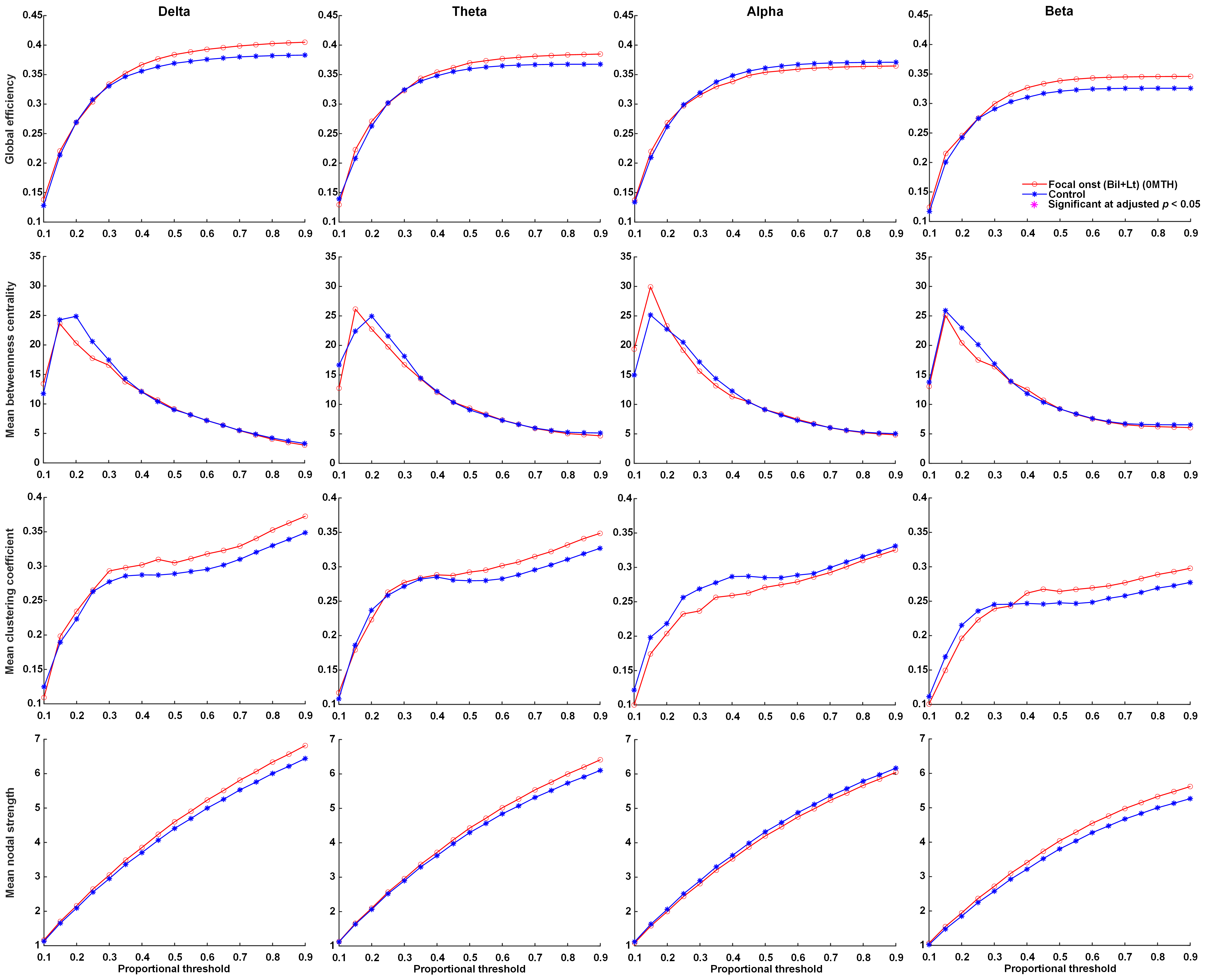

Supplement: Supplementary file 1 [file nutrients-14-04457-s001.zip › Figure S2.tif]

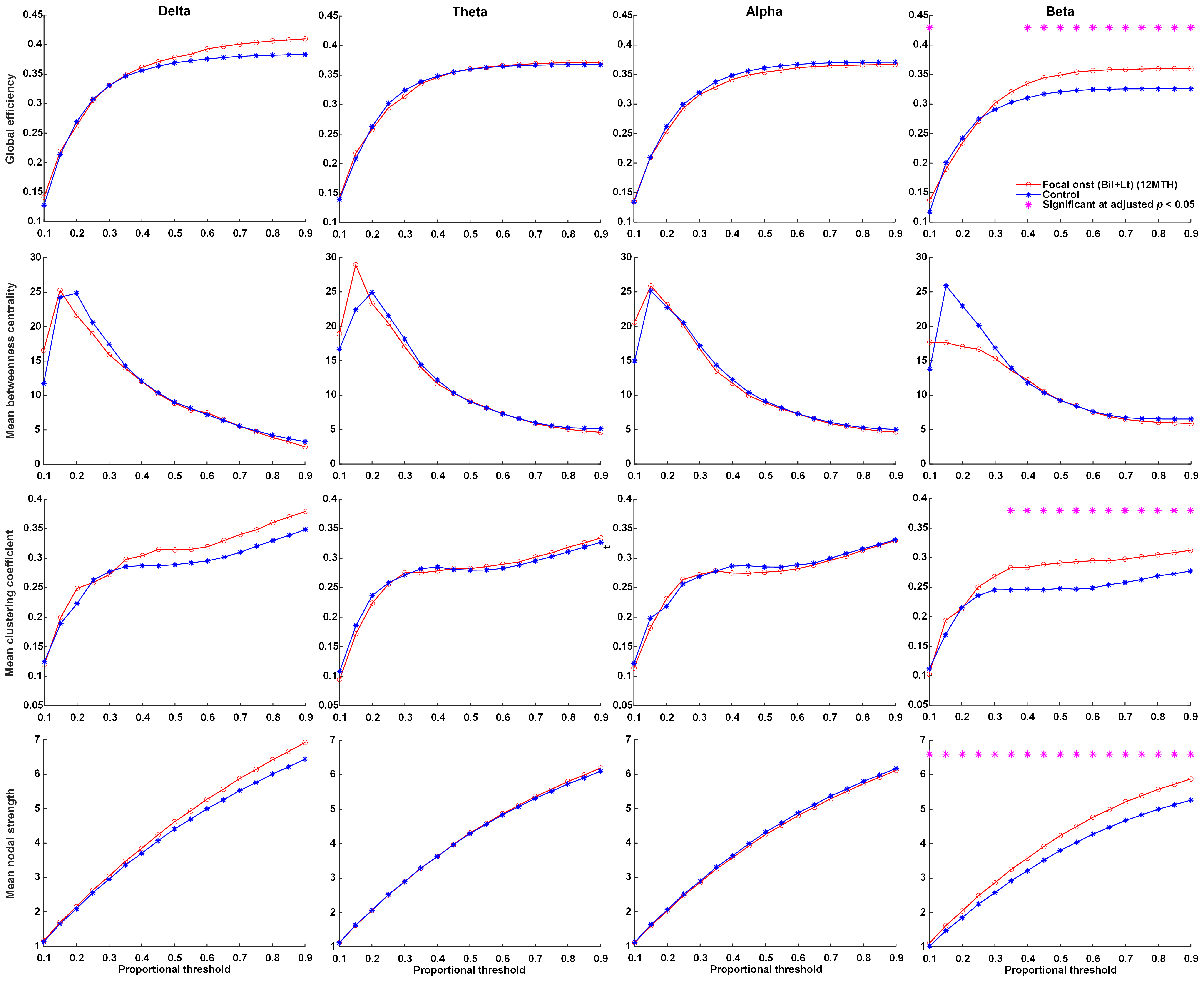

Supplement: Supplementary file 1 [file nutrients-14-04457-s001.zip › Figure S3.tif]

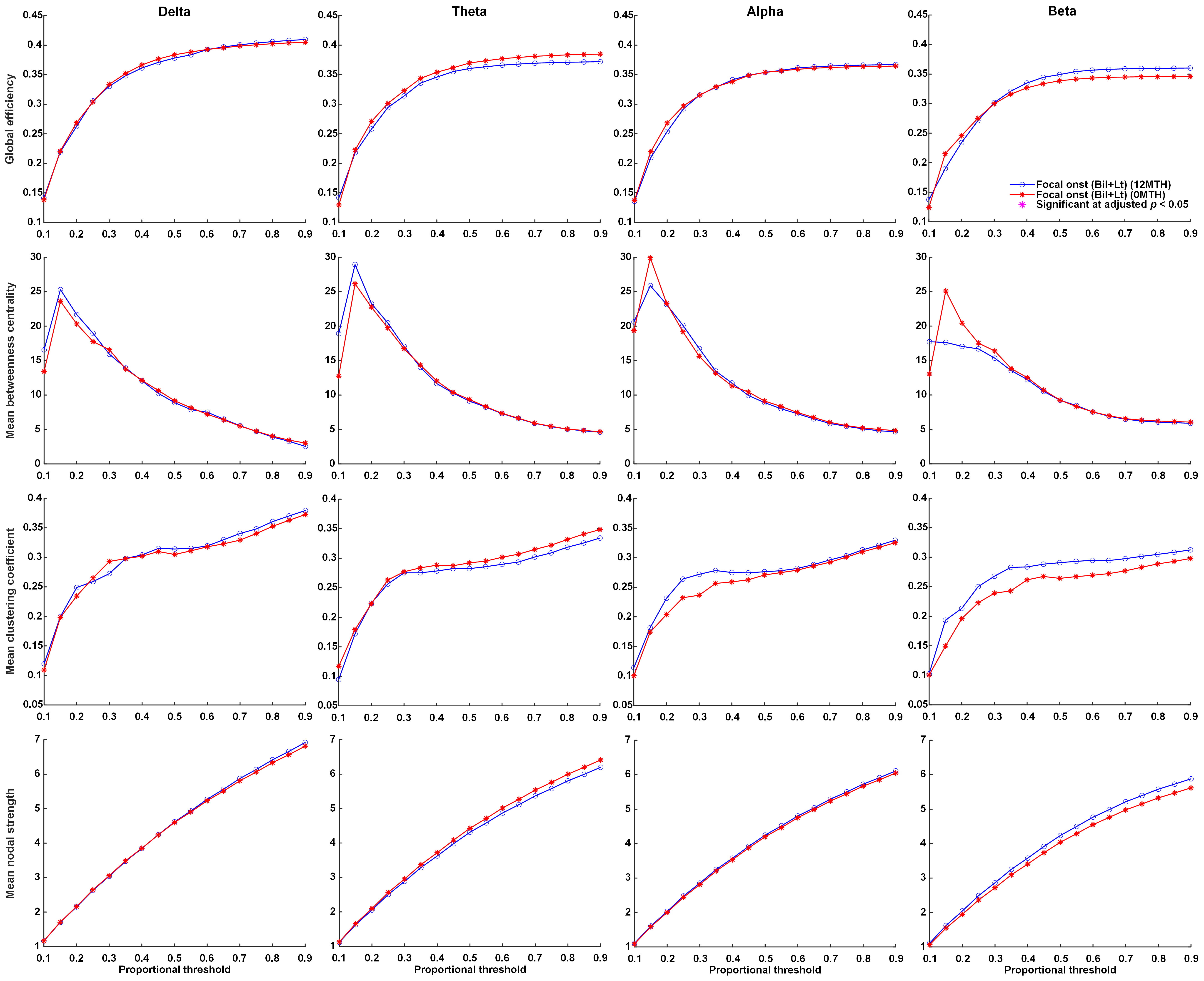

Supplement: Supplementary file 1 [file nutrients-14-04457-s001.zip › Figure S4.tif]
